# Positive selection on the K domain of the *AGAMOUS* protein in the Zingiberales suggests a mechanism for the evolution of androecial morphology

**DOI:** 10.1186/s13227-015-0002-x

**Published:** 2015-04-08

**Authors:** Ana Maria R Almeida, Roxana Yockteng, Wagner C Otoni, Chelsea D Specht

**Affiliations:** Department of Plant and Microbial Biology, University of California, Berkeley, 111 Koshland Hall, Berkeley, CA 94720 USA; Department of Integrative Biology and the University and Jepson Herbaria, University of California, Berkeley, Berkeley, CA 94720 USA; Muséum National d’Histoire Naturelle, Institut de Systématique, Évolution et Biodiversité. UMR 7205 CNRS, CP39, 16 Rue Buffon, 75231 Paris/Cedex 05, France; Current address: Corporación Colombiana de Investigación (CORPOICA), Km 14 Vía Mosquera Bogotá, Colombia; Departamento de Biologia Vegetal/BIOAGRO, Av. Peter Henry Rolfs s/n, Universidade Federal de Viçosa, Campus Viçosa, Viçosa, MG 36570-900 Brazil; Programa de Pós-Graduação em Genética e Biodiversidade, Universidade Federal da Bahia, Campus de Ondina, Salvador, BA 40170-290 Brazil

**Keywords:** *AGAMOUS*, Androecial morphogenesis, Gene duplication, K domain, Petaloidy, Positive selection, Protein divergence, Zingiberales

## Abstract

**Background:**

The ABC model of flower development describes the molecular basis for specification of floral organ identity in model eudicots such as *Arabidopsis* and *Antirrhinum*. According to this model, expression of C-class genes is linked to stamen and gynoecium organ identity. The Zingiberales is an order of tropical monocots in which the evolution of floral morphology is characterized by a marked increase in petaloidy in the androecium. Petaloidy is a derived characteristic of the ginger families and seems to have arisen in the common ancestor of the ginger clade. We hypothesize that duplication of the C-class *AGAMOUS* (*AG*) gene followed by divergence of the duplicated *AG* copies during the diversification of the ginger clade lineages explains the evolution of petaloidy in the androecium. In order to address this hypothesis, we carried out phylogenetic analyses of the *AG* gene family across the Zingiberales and investigated patterns of gene expression within the androecium.

**Results:**

Phylogenetic analysis supports a scenario in which Zingiberales-specific *AG* genes have undergone at least one round of duplication. Gene duplication was immediately followed by divergence of the retained copies. In particular, we detect positive selection in the third alpha-helix of the K domain of Zingiberales *AGAMOUS* copy 1 (*ZinAG-1*). A single fixed amino acid change is observed in *ZinAG-1* within the ginger clade when compared to the banana grade. Expression analyses of *AG* and *APETALA1/FRUITFULL* (*AP1/FUL*) in *Musa basjoo* is similar to A- and C-class gene expressions in the *Arabidopsis thaliana* model, while *Costus spicatus* exhibits simultaneous expression of *AG* and *AP1/FUL* in most floral organs. We propose that this novel expression pattern could be correlated with the evolution of androecial petaloidy within the Zingiberales.

**Conclusions:**

Our results present an intricate story in which duplication of the *AG* lineage has lead to the retention of at least two diverged Zingiberales-specific copies, *ZinAG-1* and Zingiberales *AGAMOUS* copy 2 (*ZinAG-2*). Positive selection on *ZinAG-1* residues suggests a mechanism by which *AG* gene divergence may explain observed morphological changes in Zingiberales flowers. Expression data provides preliminary support for the proposed mechanism, although further studies are required to fully test this hypothesis.

## Background

The genetic control of flower morphogenesis has long been studied in *Arabidopsis thaliana* and *Antirrhinum majus* [1]. Classically, floral organ specification has been described by combinatorial patterns of gene expression in what is well known as the ABC model of floral organ identity. In this model, specific domains of expression of A-, B-, and C-class MADS-box genes correlate with the position of the developing sepals (A-class genes only), petals (a combination of A- and B-class genes), stamens (a combination of B- and C-class genes), and gynoecium (C-class genes only): thus, gene expression is correlated with organ identity. In *A. thaliana*, there are two A-class genes (*APETALA-1* (*AP1*), and *APETALA-2* (*AP2*)), two B-class genes (*APETALA-3* (*AP3*), *PISTILLATA* (*PI*)), and one C-class gene (*AGAMOUS* (*AG*)). With the exception of *AP2*, all other genetic components of the ABC model are type-II MIKC^c^ MADS-box genes as determined by their arrangement of protein domains. Their proper function as transcriptional regulators is dependent on the protein-protein interactions that occur between the A-, B-, and C-class genes, as well as with the *SEPALLATA* genes [2], to form protein dimers and functional tetramers. The protein-level explanation for A-, B-, and C-class functions is known as the quartet model, and asserts that only in tetramers are the A-, B-, and C-class proteins capable of regulating downstream genes (for review, [3]).

Although the ABC model has proven fruitful in describing organ identity and floral organ development, it lacks a mechanism to explain how such robust gene expression patterns are established during development and how changes in expression, function, or copy number may correlate with evolutionary changes in organ morphology. In order to address this mechanism, the A-, B-, and C-class genes have been integrated into an elegant complex-system approach, capable of explaining the robustness of the ABC gene expression patterns during flower development [4,5]. By mapping the landscape of known gene interactions during floral development in *A. thaliana*, Mendoza and coworkers (1999) were able to recover the stable states (that is, attractors) that correspond to the gene expression patterns correlated to floral organ identity, as described by the ABC model. In doing so, the authors proposed a set of necessary and sufficient genes and genetic interactions that provide a dynamical and mechanistic explanation for the establishment of the ABC gene expression patterns [6,7].

**Figure 1:**
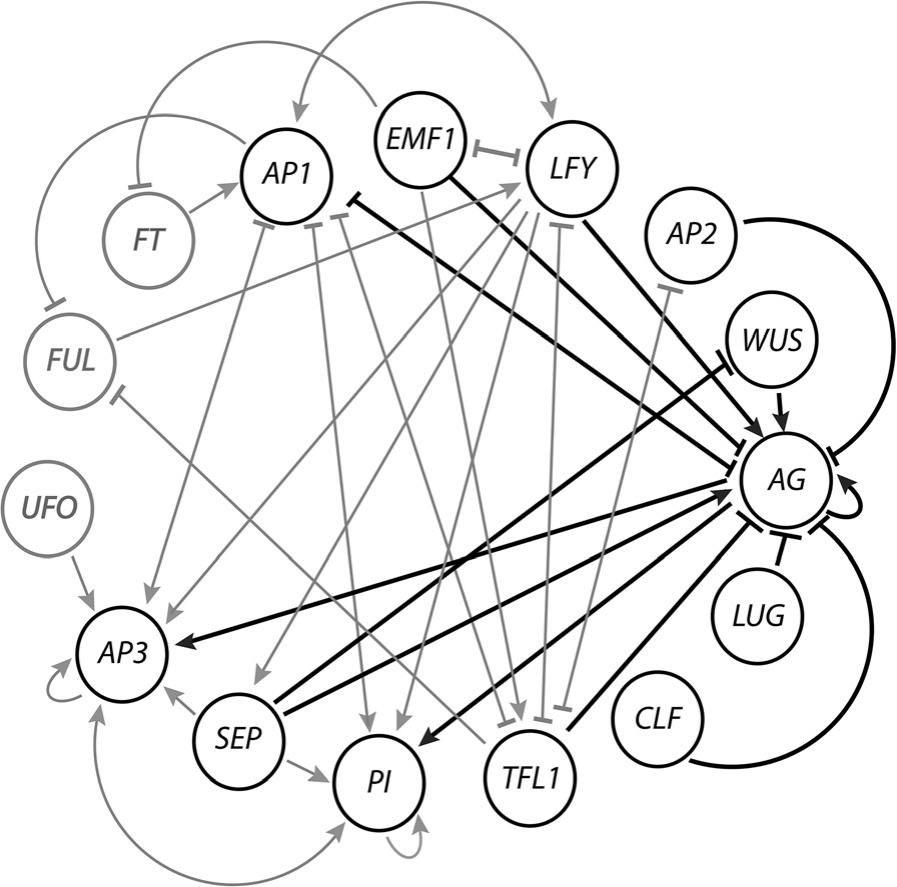
**Floral organ specification gene regulatory network (FOS-GRN), modified from Álvarez-Buylla, E.R** ***et al.*** [6]). The circles (nodes) represent genes or proteins that are experimentally shown to participate in floral organ specification during flower morphogenesis in *Arabidopsis thaliana*. Arrows indicate positive interactions between nodes, while the T symbols indicate negative interactions between nodes. Fifteen genes are depicted: *EMF1*, *LFY*, *AP2*, *WUS*, *AG*, *LUG*, *CLF*, *TFL1*, *PI*, *SEP*, *AP3*, *UFO*, *FUL*, *FT*, and *AP1*. Direct interactions between *AGAMOUS* (*AG*) and other genes in the FOS-GRN are highlighted in black. Other interactions within the FOS-GRN not directly involving *AG* are depicted in gray, as well as genes that are not direct interactors with *AG*.

Within the mapped gene interactions that constitute the floral organ identity gene regulatory network (FOS-GRN, Figure 1), *AGAMOUS* is one of the most highly interconnected genes, suggesting that alterations in this node are likely to constitute important changes in the stable states of the system, thereby functioning as a potential nexus for evolutionary change. In particular, *AG* may be a key regulator of androecial (stamen) morphology; stamen and petal stable gene expression patterns (also known as GRN “attractors”) in *A. thaliana* differ exclusively by opposite states of *AP1* and *AG* expression in which *AP1* is expressed in petals but not in stamens and *AG* is expressed in stamens but not in petals [8]. Thus, we propose that an understanding of the evolution of the *AG* family across Zingiberales, especially comparing the expression patterns of *AG* and determining its potential for interactions with *AP1*, can provide insight into the evolution of petaloidy in the stamen whorl.

The *AG* gene was first isolated from *A. thaliana* over two decades ago [9], when fully penetrant mutations were shown to cause abnormalities in the development of the floral reproductive organs. *AG* has since been implicated in proper development of reproductive organ identity across flowering plants and is thought to play an additional role in ovule development and meristem determinacy in some lineages [10-12]. The evolution of the *AG* subfamily of transcription factors has been extensively studied across angiosperms. Phylogenetic analyses of the *AG* subfamily demonstrate that a duplication event occurring early during angiosperm diversification resulted in the origin of two major lineages: the *AG* and the *AGAMOUS-like 11* (*AGL11*) lineages [13-16]. In the *Arabidopsis* canonical model, the *AG* and *AGL11* lineages correspond functionally to C-class and D-class homeotic genes, respectively, in which C-class homeotic genes are involved in stamen and carpel identity and in floral meristem determinacy and D-class genes are more specifically involved in ovule and fruit development [14,12]. The *AG* lineage itself has undergone subsequent gene duplications, and parsing of function of duplicated copies is thought to have occurred independently in the major angiosperm lineages. Within the core eudicots, for example, the *AG* lineage is divided into the *euAG* and the *PLENA* (*PLE*) lineages [14,15] with various subclades within both lineages demonstrating neofunctionalization, subfunctionalization, redundancy, and loss of duplicated copies [17]. In *Antirrhinum*, the *AG* lineage genes *PLE* and *FARINELLI* (*FAR*) contribute unequally to specify male and female reproductive organs [18,19], while in *Petunia FBP6* and *PMADS3* act redundantly as C-function genes [20]. In *Zea mays*, however, the *AG* paralogs *ZAG1* and *ZMM2* appear to be expressed in spatially distinct domains of the developing flower, and may have subfunctionalized into carpel- and stamen-specific paralogs [21], while in *Oryza sativa*, *AG* paralogs *OSMADS3* and *OSMADS58* are essential for reproductive organ identity and together with *AG11* lineage *OSMADS13* are important for floral meristem determinacy [10,12,13].

In addition to its role in reproductive organ identity, differential expression of *AG* in *A. thaliana* has been shown to be involved in the development of petaloidy in the androecium. For instance, the *ag-11* allele, bearing a single point mutation in the regulatory region of *Arabidopsis AG*, results in the transformation of stamens into petaloid organs [22]. Also, downregulation of *AG* by anti-sense RNA in *A. thaliana* leads to a variety of aberrant floral morphologies, including petaloid stamens [23]. Most recently, Tanaka *et al*. [24]) demonstrated the role of *AG* in regulating proper petal and stamen differentiation in cyclamen (*Cyclamen persicum*), where two paralogs (*CpAG1* and *CpAG2*) are present and repression of *CpAG2* leads to the formation of infertile and petaloid structures in the stamen whorl [24].

The molecular mechanisms underlying the evolution of androecial petaloidy in the angiosperms have not been studied in detail. It is likely that developmental processes underlying androecial petaloidy are homoplasious across flowering plants, as petal-like stamens have evolved independently in a variety of angiosperm lineages [25]. In male flowers of the early diverging *Amborella trichopoda*, stamen filaments are expanded into petal-like structures. In several other basal angiosperm and magnoliid lineages, flowers display a gradual transition between petal and stamen organs, with multiple degrees of androecial petaloidy present even within the same flower (for example, *Nymphaea alba*) [26]: A gradient of *AG* and B-class gene expression has been implicated in this gradual morphological transition between laminar petals and filamentous stamens [27].

**Figure 2:**
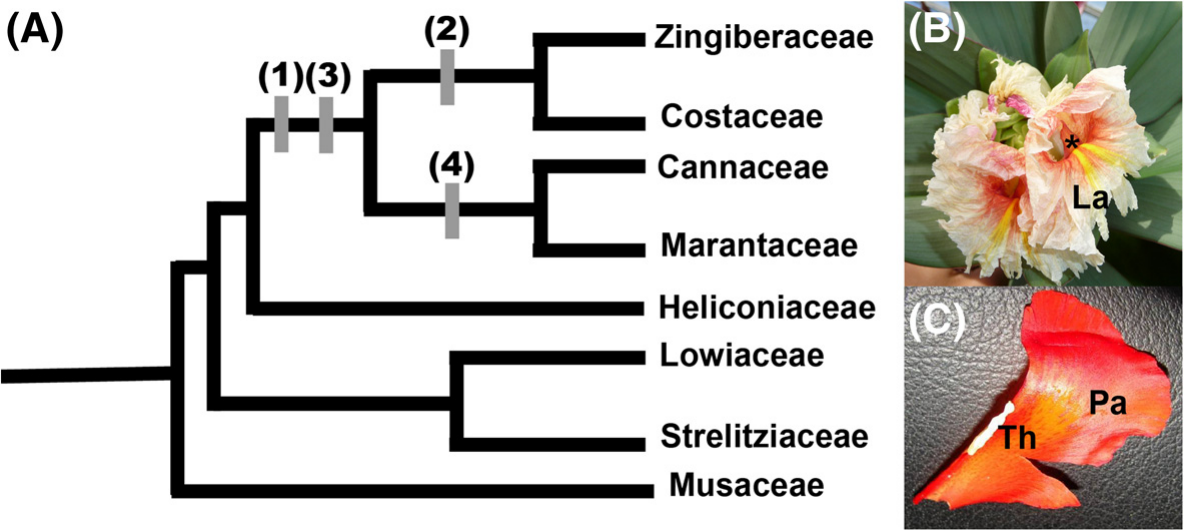
**Phylogeny of Zingiberales with key events in androecial evolution. (A)** Morphological character states of the androecium are mapped onto the most recent Zingiberales phylogeny [28]. The eight Zingiberales families are divided into two groups: the first diverging banana lineages (Heliconiaceae, Strelitziaceae, Musaceae, and Lowiaceae), and the derived ginger clade (Zingiberaceae, Costaceae, Marantaceae, and Cannaceae). Main changes in androecial morphology are depicted with numbers. (1) Reduction in the number of fertile stamens, from 5 to 6 fertile stamens in the banana lineages, to 1 fertile stamen in Zingiberaceae and Costaceae or ½ of a fertile stamen in Marantaceae and Cannaceae; (2) fusion of petaloid staminodes leading to the formation of the labellum. Five infertile stamens fuse in Costaceae, while 2 or 4 staminodes form the labellum in the Zingiberaceae; (3) laminar extension of the filament of the fertile stamen; (4) abortion of a theca of fertile stamen. **(B)** *Costus* sp. flowers. (La) labellum; Asterisk indicates the abaxial side of laminar connective of fertile stamen. **(C)** *Canna indica* one half fertile stamen. (Th) single theca; (Pa) petaloid appendage resulting from the laminar expansion of the filament [29].

In order to further explore the role of the *AG* gene in the evolution of androecial petaloidy across angiosperms, we focus our study on the Zingiberales, a group of monocots that exhibits extensive petaloidy in the androecial organs. Zingiberales are an order of tropical monocots comprising approximately 2,500 species. The order is divided into eight families, traditionally organized into the paraphyletic banana families (Musaceae, Lowiaceae, Strelitziaceae, and Heliconiaceae) and the derived ginger clade (Zingiberaceae, Costaceae, Cannaceae, and Marantaceae) ([28], Figure 2). In the Zingiberales, androecial petaloidy is an important component of floral morphological diversity: lineages of the ginger clade have a marked reduction in the number of fertile stamens. The staminodes (infertile stamens) develop as petal-like structures and usually constitute the bulk of floral display, with the petals of the same flower developing as relatively inconspicuous structures in comparison with the stamen whorl (Figure 2). In Costaceae and Zingiberaceae, 2 to 5 of the petaloid staminodes fuse together to form a novel organ, the labellum.

Given the involvement of the *AG* gene lineage in reproductive organ development and its interconnectivity within the floral organ specification gene regulatory network (FOS-GRN), we hypothesize that gene duplication followed by potential functional divergence of the *AG* lineage in the ginger clade is correlated with the evolution of petaloidy in the androecium. In order to test our hypothesis, *AG* lineage genes were amplified from across the Zingiberales, and their expression was assessed during flower development in representative species. Tests of selection were carried out to investigate the role of selection on gene evolution and function. Our results suggest that positive selection has played a role in the evolution of *AG* across the Zingiberales order, particularly in protein divergence within the K domain. These protein modifications, together with comparative analyses of *AG* and *AP1* expression across the order, suggest a mechanism by which androecial petaloidy may have evolved in the Zingiberales, and support the hypothesis that modifications in *AG* expression and function are correlated with androecial petaloidy.

## Methods

### Plant material, RNA extraction, and cDNA synthesis

Twenty species from seven of the eight Zingiberales families were sampled in order to represent the diversity of floral form observed in the order (Table 1). Fresh flowers were collected and stored in a homemade recipe equivalent of “RNA-later” for up to 2 weeks prior to RNA extraction. Total RNA was extracted from floral material using Plant RNA Extraction Reagent (Invitrogen, Carlsbad, CA, USA), according to Yockteng *et al*. [30]. RNA was stored at −80°C until further use. Prior to cDNA synthesis, RNA was treated with DNAse (Fermentas). cDNA synthesis was performed using iScript select cDNA synthesis Kit (Bio-Rad, Hercules, CA, USA) and polyT primers. Amplification of the ß-actin gene as a positive control for cDNA synthesis was performed using PCR primers (F: 5′ GGA CGA ACA ACT GGT ATC GTG CTG 3′ and R: 5′ GAT GGA TCC TCC AAT CCA GAC ACT GTA 3′) [31]. Reactions without reverse transcriptase (no-RT) were used as negative control.

**Table 1:** **List of species and accession numbers used in this study**

**Family**	**Species**	**Collection**	**NCBI accession**
Musaceae	*Musa acuminata*		EU869310.1; DQ060444.1
	*Musa basjoo*	UCBG-89.0873	
	*Musa velutina*	L-67.0284	
Strelitziaceae	*Phenakospermum guyanense*	PTBG 047865	
	*Strelitzia reginae*	UC-MB0607	
Heliconiaceae	*Heliconia pendula*	McB-711003-003	
Costaceae	*Costus spicatus*	NMNH-2002-127	
	*Costus products*	UCBG-2009.0525	
	*Monocostus uniflorus*	UCBG-1994.725	
Cannaceae	*Canna jaegeriana*	UC-MB0854	
Zingiberaceae	*Globba laeta*	L-92.0182	
	*Alpinia pinetorum*	UC-MB0835	
	*Elettariopsis smithiae*	L-93.0137	
	*Zingiber rubromarginata*	UC-MB0876	
	*Alpinia oblongifolia*	UC-MB0835	
	*Kaempferia rubromarginata*	L-2003.0153	
	*Alpinia oblongifolia*		DQ286724.1
Marantaceae	*Monotogma guianense*	L-78.1340	
	*Stachyphrynium jagoranium*	L-2003.0192	
	*Ischnosiphun helenae*	UC-MB0844	
	*Calathea princeps*	UC-MB0863	
Poaceae	*Zea mays*		NM_001111851.1; NM_001111456.1; X80206.1; X81199.1
	*Oryza sativa*		L37258.1; NM_001061424.1; AF151693.1
	*Hordeum vulgare subsp. Vulgare*		AF486648.1
Arecaceae	*Elaeis guineensis*		AY7399.1; AY739698.1
Triuridaceae	*Lacandonia schismatica*		GQ214163.1
Asparagaceae	*Hyacinthus orientalis*		AF099937.1
Liliaceae	*Lilium longiflorum*		AY829227.1
Orchidaceae	*Phalaenopsis equestris*		JN983500.1
	*Dendrobium thyrsiflorum*		DQ017703.1
Hydrangeaceae	*Hydrangea macrophylla*		AB453919.1
Rosaceae	*Prunus serotina*		EU938540.1
Brassicaceae	*Arabidopsis thaliana*		AY727648.1; AY727647.1; AY727624.1; NM_001203767.1
Magnoliaceae	*Magnolia odoratissima*		JQ326240.1; JQ326255.1

PTBG: Pacific Tropical Botanical Garden; L: Lyon Arboretum, Oahu, Hawaii; UC: University of California at Berkeley Herbarium; UCBG: University of California Botanical Garden; McB: McBryde Botanical Garden, Kauai, Hawaii; NMNH: Smithsonian Greenhouses.

### Amplification of *AGAMOUS* genes in the Zingiberales

A multiple sequence alignment (MSA) for the *AG* gene lineage was generated from sequences downloaded from NCBI (Table 1). The MSA was used to design general primers for amplification of Zingiberales *AG* genes. Multiple primer combinations, with different degrees of degeneracy, were used in order to improve chances of assessing all copies of the *AG* gene lineage within the Zingiberales. Primer sequences were as follows: (i) forward primers: 5′ ACI AAY MGI CAR GTI ACI TTY TG 3′; 5′ ATG GSI MGI GGI AAR ATI SAR AT 3′; 5′ CAR GTK ACC TTC TGC AAG 3′; 5′ ATC CCA TGG AGC ATA AAG CA 3′; 5′ GRG GRA AGA TCG AGA TCA AG 3′; (ii) reverse primers: 5′ ACC CTA TCA GTC TCG GCG ATC TTG TTC C 3′; 5′ TCA TCG TTC AAC CAA AGT GG 3′; 5′ TTG MAK RAA GTT CCY TGA RTM RT 3′.

PCR reactions were carried out using Phire Hot Start II DNA Polymerase (Thermo Scientific) and 2 μl of 5X Phire buffer; 0.2 μl 10 mM dNTPs; 0.5 μl of each primer; 0.1 μl Phire Polymerase; 1 μl [1:10] cDNA; and ddH_2_O, for a total volume of 10 μl. Thermocycling conditions followed manufacturer’s recommendations. PCR products were visualized on a 1% agarose gel stained with GelRed™ (Phoenix Research Products) according to the manufacturer’s protocol. PCR products were cloned into Top10 cells and sequenced using Big Dye Terminator Kit v3.1 (Applied Biosystems) on a 3700 sequencer. At least 16 clones were sequenced for each of the species sampled. Over 40 clones were sequenced for *Costus spicatus*, in order to insure deep sampling of gene copy number in this species.

### Phylogenetic analyses

A multiple sequence alignment was generated using MacClade (4.06 OS X) with all generated Zingiberales sequences aligned to outgroup sequences downloaded from NCBI (Table 1). Model selection for the final alignment was tested in jModeltest 0.1.1 [32] using the Bayesian information criterion (BIC). PartitionFinder [33] was used to test for the best partition scheme for the dataset and substitution model, also based on the BIC criterion. According to PartitionFinder as well as jModelTest, the best-fit model was K80 + G and no data partition was advised. The best-fit model was implemented in MrBayes 3.2 [34] and PhyML [35] in order to assess gene tree topology. MrBayes runs were implemented on the CIPRES Science Gateway (www.phylo.org) under the model specified above, as well as under variations of the best-fit model to ensure that topology was not influenced by model selection and ran for 1.5 M generations. Data were further analyzed for convergence with Tracer v1.5 (http://beast.bio.ed.ac.uk/). SumTrees v.3.0.0 using the DendroPy Phylogenetic Computing Library v3.7.1 [36] was used to calculate the burn-in and remove the appropriate trees saved prior to stationarity, and to assemble a 50% majority rule tree from the remaining trees. Maximum likelihood (ML) analyses were performed using PhyML implemented on the ATGC South of France bioinformatics platform (http://atgc.lirmm.fr/phyml/). Bootstrap support from 100 replicates and posterior probabilities (PP) were calculated for ML and MrBayes analyses, respectively, and are used as branch support in the gene tree (bootstrap/PP). In order to test the likelihood of different evolutionary scenarios for the *AG* gene tree, a Shimodaira-Hasegawa (SH) test [37] was performed by manually generating a constrained gene tree in which the two first branching lineages (all “*ZinAG-1*”) were forced to form a monophyletic group. The likelihood of the constrained tree was tested against the unconstrained gene tree obtained in our phylogenetic analysis using the likelihood ratio test (LRT). Likelihood tests for constrained topologies were obtained in PAUP* [38].

### Selection tests

Both branch- and site-specific selection tests were performed in order to assess signals of selection across the *AG* subfamily, along branches leading to the major clades and at specific amino acid sites. Branch selection was assessed using PAML *codeml* branch models by setting the model = 2 in order to allow several omega (*w*) ratios compared to a fixed *w*, while site selection was evaluated using the site models M1a and M2a [39]. Site selection was also assessed using the fixed-effect likelihood (FEL) model in HyPhy 2.0 [40] under a stringent cut-off of 0.1, as suggested by the HyPhy program. A Bayesian 50% majority rule consensus tree was used for all selection analyses. For each node tested, a two-rate analysis was used to allow adjustment of the ratio of non-synonymous (dN) and synonymous (dS) substitutions across sites, and models determined by jModeltest were specified for the nucleotide model of evolution.

### Gene expression of *AGAMOUS* and *APETALA1* in Zingiberales developing flowers

RT-PCRs for *ZinAG-1* and *ZinAG-2* were carried out in all *Musa basjoo* floral organs. Primers were designed on intron-exon boundaries whenever possible, and sequences are as follows: *MbAGcp1* forward 5′ TTG AAA GGT ATA AGA AAG CAT 3′; *MbAGcp1* reverse 5′ TTA TTC TCG AGT TGC TTC ATG TCT 3′; *MbAGcp2* forward 5′ TCG AGA GGT GGT ACA AGA AAG CAT GT 3′; *MbAGcp2* reverse 5′ CGA GTC TCA AGC TGC TTC AG 3′. Reactions were carried out using Phire Hot Start II DNA Polymerase (Finnzymes, Finland) and the following protocol: 2 μl of 5X PHIRE buffer; 0.5 μl of each of the 10 mM primers; 0.1 μl of PHIRE polymerase; 0.2 μl of 50 mM dNTPs; 1 μl of [1:10] dilution of organ-specific cDNA; and water up to a 10 μl total volume, for 25 cycles. PCR reactions were performed on BioRad Thermocyclers and were visualized on 1% agarose gel, post-stained with Gel-Red™ (Biotium).

Expression of *AGAMOUS* and *APETALA1* in *C. spicatus* and *M. basjoo* was assessed by generating organ-specific transcriptomes. cDNA libraries for sequencing on the Illumina platform were prepared using the TruSeq RNA sample prep kit v2. Two cDNA libraries each were prepared with 2.0 μg of RNA extracted from dissected tissue of the filament, theca, and free petal of *M. basjoo* and the petaloid filament, theca, petal, and labellum (fused petaloid staminodes) of *C. spicatus*. Libraries were multiplexed using barcoding set A. Samples were run on a HiSeq2000 at IIGB HT Sequencing Facility at the University of California, Riverside. Raw reads were trimmed to remove adapters and regions of poor quality with *cutadapt* [41]. *Costus spicatus* sequences were assembled into a reference transcriptome using Trinity [42] with minimum contig length of 300. All other parameters were used according to Trinity default settings. GMAP/GSNAP [43] was used to align *M. basjoo* trimmed reads to annotated CDS from the published *Musa acuminata* genome, while *C. spicatus* trimmed reads were aligned to the *C. spicatus* reference transcriptome. Expression of *AG* and *AP1-like* genes was estimated using eXpress [44] in units of FPKM (frequency per kilobase of exon per million aligned reads). Replicates were independently processed, and gene expression was compared between libraries for consistency. *ACTIN1* expression was used to normalize targeted gene expression across transcriptome libraries. Error bars were calculated based on the standard deviation (SD) of the two normalized samples for each organ.

In order to confirm transcriptome expression data for *C. spicatus AG* and *AP1-like* genes, quantitative PCR was performed on each floral organ of *C. spicatus* flowers (sepals, petals, labellum, stamen, and gynoecium). A general *C. spicatus AP1/FUL*-like primer was designed, as at this point, we were not able to produce copy-specific primer pairs. qPCR was performed on *C. spicatus* floral organs using iQ™ SYBR® Green Supermix (Life Science Research), and following the manufacturer’s protocol. Clade-specific qPCR primers were designed based on the nucleotide differences between *C. spicatus* sequences from different clades on the *AGAMOUS* gene tree, and primer sequences are as follows: *CsAGcp1* forward 5′ AAC AGC AGT GTG AGA GCG ACT 3′; *CsAGcp1* reverse 5′ GGT CTC TAA GGC TCA TAG AAC CGA GA 3′; *CsAGcp2* forward 5′ CCA ACA GTG TGA GAG CAA CAA 3′; *CsAGcp2* reverse 5′ CTT CAT ATC GCG TAG GCT CA 3′; *CsAP1/FUL-like* forward 5′ ATA TCA GGT CAA GAA AGA ACC AAA TC 3′; *CsAP1/FUL-like* reverse 5′ GGG CTT GTT TGG ATT CGT T 3′. Two reactions were performed, one for *ZinAG-1* and *ZinAG-2*, and another for *AP1/FUL* and ZinAG-1 with annealing temperatures of 60°C and 58°C, respectively. In both cases, three replicates per gene were performed and *ACTIN1* was used as an internal control.

## Results

### Amplification and phylogenetic analyses

*AG* sequences were obtained for all families within the order, with the exception of Lowiaceae (*Orchidantha*), a monogeneric lineage. A multiple sequence alignment (MSA) of 558 bp was generated and encompasses all protein domains, with the exception of the first nine codons of the MADS domain and the end of the C-terminal domain, for which alignment to outgroup sequences became increasingly challenging. The final MSA comprises a total of 37 ingroup and 13 outgroup sequences. This MSA was used as the input to jModelTest, and determined the best-fit model as the K80 + G model. The best-fit model, as well as other more parameterized models (GTR, GTR + I, and GTR + I + G), was implemented in both MrBayes and PhyML.

**Figure 3:**
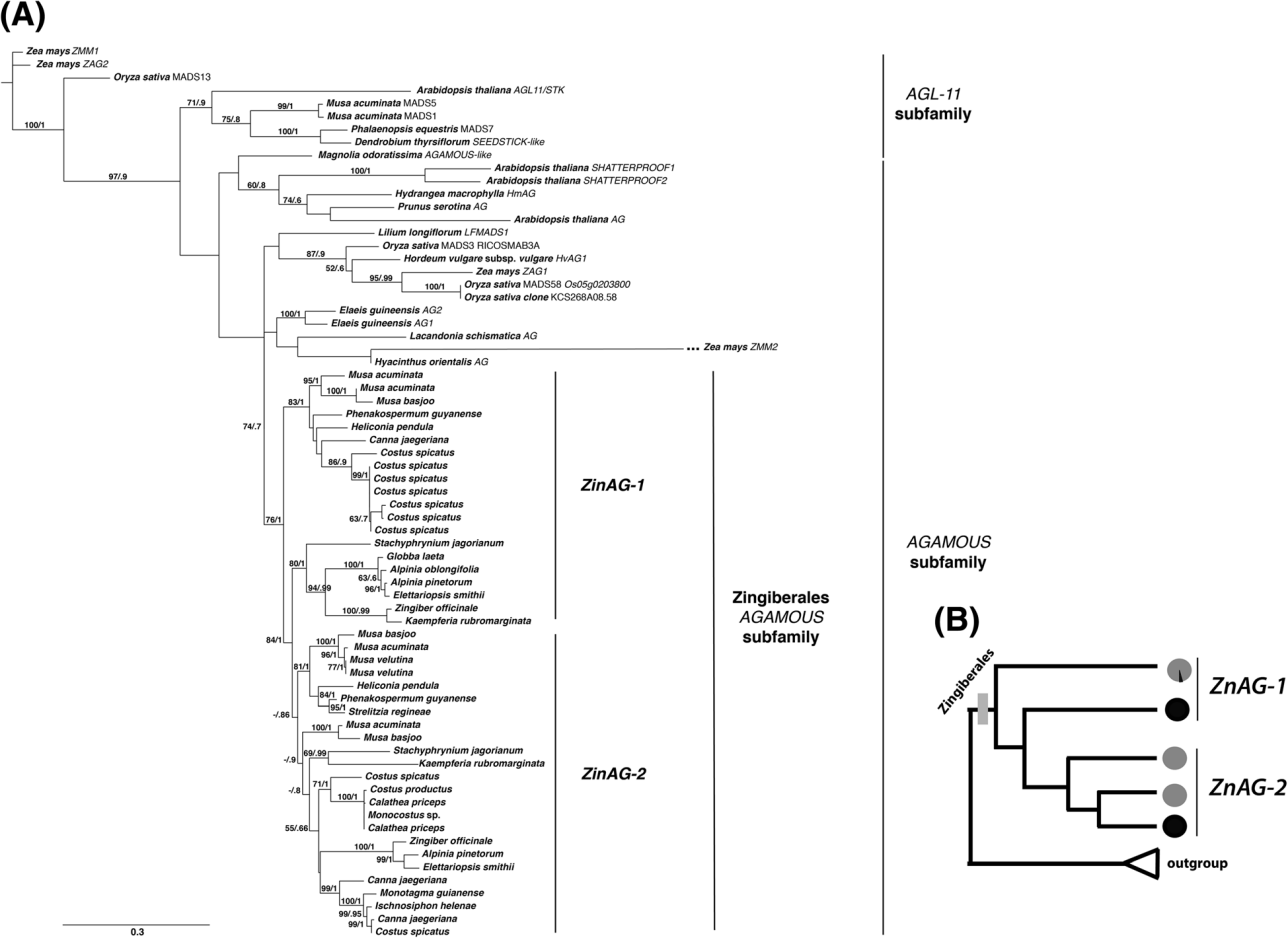
***AGAMOUS*** **(** ***AG*** **) gene tree for the Zingiberales. (A)** Bayesian topology generated in MrBayes with 1.5 M generations, under the K80 + G model (best fit model according to jModelTest). The general tree topology agrees with results generated by maximum likelihood (ML) analysis (PhyML), as well as under different models for both Bayesian and ML analyses. Partition of the data set according to codon position rendered an unresolved tree with poor likelihood (data not shown). Only bootstrap support >50% are presented, followed by posterior probabilities (PP). At least two copies of the gene *AG* can be identified, according to the distribution of ginger clade and banana grade species in the gene tree. **(B)** Schematic representation of the *AG* gene tree. Black circles represent sequences from the ginger clade, while gray circles represent sequences from the banana grade. Each circle indicates the position of the corresponding sequences in the gene tree. This schematic tree depicts one clade (*ZinAG-2*) comprising ginger clade and banana grade sequences, and two early branching lineages comprising banana grade and ginger clade sequences (*ZinAG-1*).

Tree topology across methods and models was largely congruent (Figure 3). All *AG* sequences from Zingiberales form a monophyletic group with high support (76% bootstrap and posterior probability of 1). According to the species distribution on the gene tree, there are at least two copies of the *AG* gene in the Zingiberales, herein called *ZinAG-1* and *ZinAG-2* (Figure 3B). *ZinAG-2* sequences form a monophyletic group, suggesting an orthologous relationship between copies found in the banana and ginger lineages, while *ZinAG-1* sequences formed a grade at the base of the *ZinAG-2* clade.

**Figure 4:**
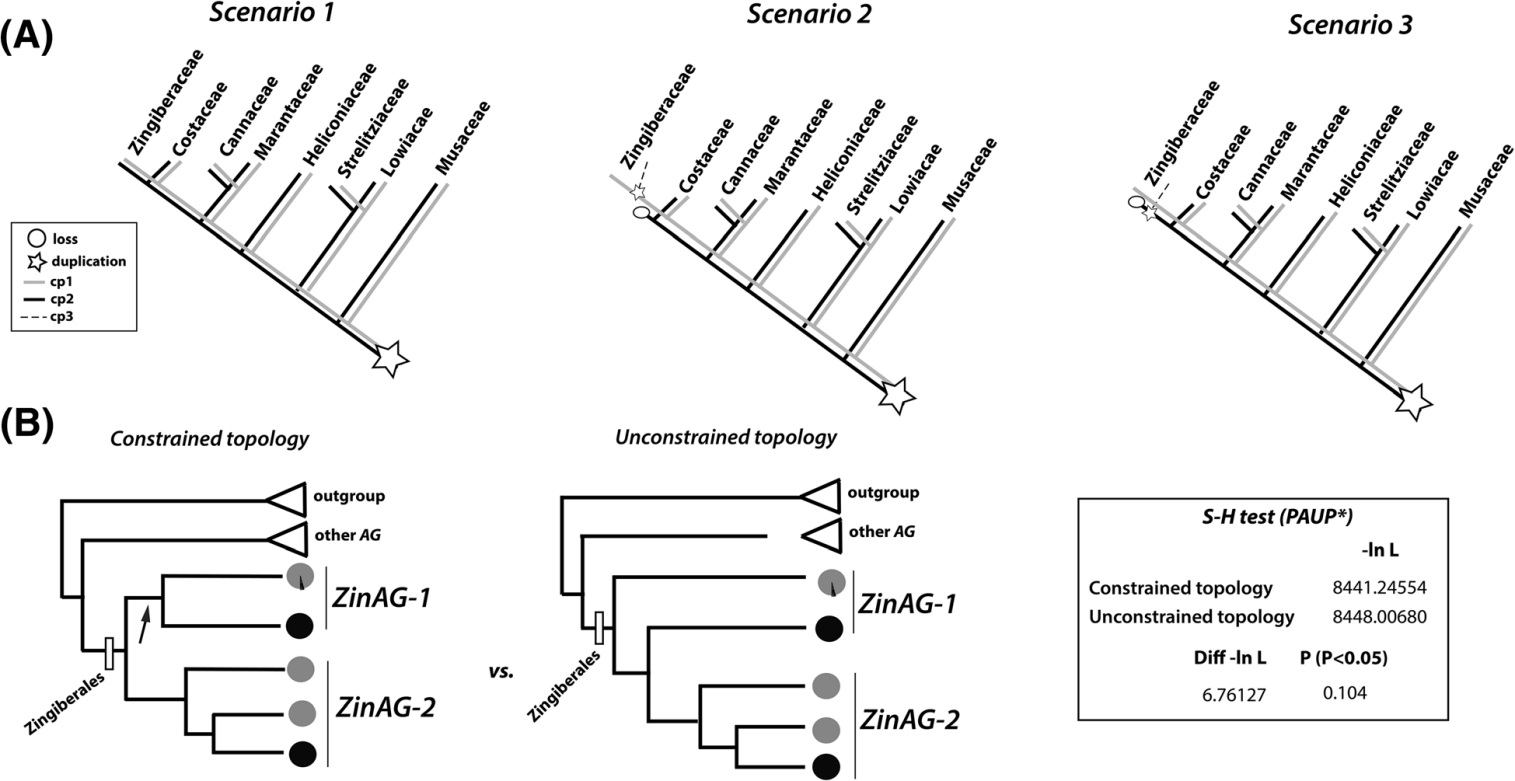
**Evolution of the** ***AGAMOUS*** **gene lineage. (A)** Potential AGAMOUS (AG) gene copy histories within the Zingiberales. Scenario 1 assumes one single duplication event at the base of the Zingiberales order, leading to two distinct orthologous AG lineages (ZinAG-1 and ZinAG-2). Scenarios 2 and 3 depict alternative histories of duplications and losses of the AG copies, particularly in the Zingiberaceae lineage. In both cases, orthologous relationships would be complicated by the existence of subsequent duplication events, unique to the Zingiberaceae lineage, leading to the evolution of yet another copy of AG, ZinAG-3. **(B)** Shimodaira-Hasewaga test (Shimodaira & Hasewaga [37]), SH test) was performed using PAUP* on a constrained topology, where the two first paraphyletic lineages were forced to form a monophyletic clade. The likelihood score for the constrained topology was compared to the likelihood score of the unconstrained gene tree, as obtained on Bayesian and maximum likelihood phylogenetic analysis (Figure 3). The constrained topology shows a better likelihood score than the one calculated for the gene tree topology presented here (although not significantly different), supporting the idea that the first two paraphyletic lineages are actually derived from a single duplication event. This result supports the evolutionary history depicted by Scenario 1, in which ZinAG-1 and ZinAG-2 are orthologous lineages.

According to the *AG* gene tree, several different evolutionary scenarios could account for the recovered topology for Zingiberales *AG* phylogeny (Figure 4A). Although *ZinAG-1* sequences appear paraphyletic based on the recovered topologies, these sequences could result from a single duplication event predating the divergence of the Zingiberales that ultimately resulted in two clades: *ZinAG-1* and *ZinAG-2* (Figure 4A, scenario 1). Differential sequence divergence resulting from distinct evolutionary pressures on *ZinAG-1* could result in an unresolved clade of copy 1 sequences, with phylogenetic analyses resolving a paraphyletic grade. In this case, the phylogenetic analysis would recover incongruence between the gene tree (placing the sequences as paraphyletic rather than within a single clade) and the organismal tree. Alternatively (Figure 4A, scenarios 2 and 3), a second duplication event may have occurred only in the Zingiberaceae lineage after it diverged from Costaceae, leading to a third lineage-specific *AG* copy (*ZinAG-3*) in the Zingiberaceae. This copy was retained while the paralogous duplicate was subsequently lost from the Zingiberaceae, yielding only two copies but with less clear orthology to the two copies found in the remaining Zingiberales lineages.

The SH test [37] was performed using a constrained gene tree in which the two first branching lineages (all “*ZinAG-1*”) were forced to form a monophyletic group (Figure 4B), and compared for likelihood score against the unconstrained gene tree obtained in our analyses (Figure 3). Our results indicate that the constrained gene tree has a likelihood score that is not significantly different from the unconstrained analysis, suggesting that scenario 1 (Figure 4A) is equally as likely as scenario 2 in describing the evolutionary history for *ZinAG*. The most parsimonious explanation, then, is described by scenario 1, in which a single duplication event happened at the base of the Zingiberales order prior to lineage diversification.

### Selection tests

**Figure 5:**
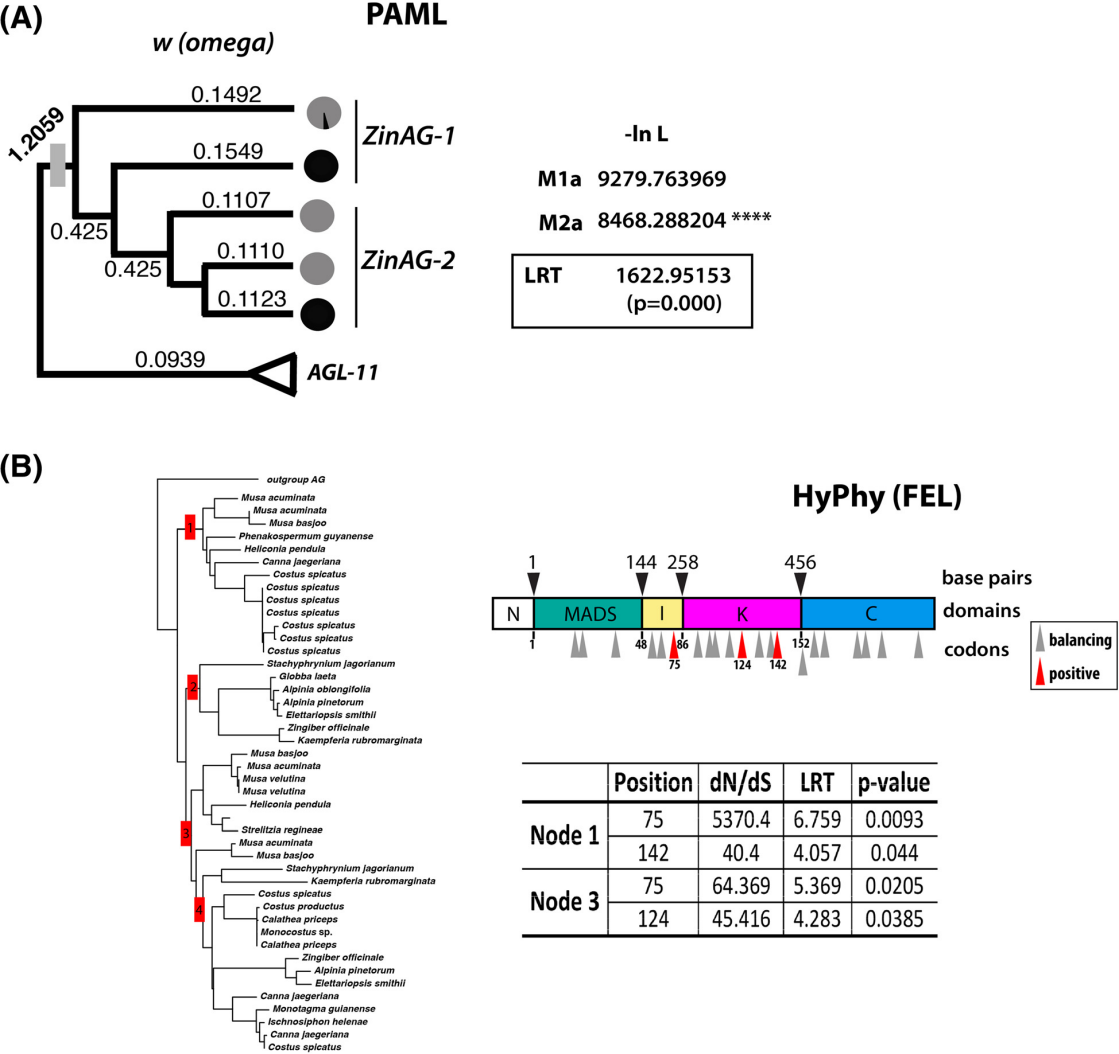
**Selection test results. (A)** PAML branch selection test. Omega (*w*) values are depicted for each branch in the gene tree. A likelihood ratio test (LTR) for branch models (M1a and M2a) was performed. PAML detects a strong selection signal at the base of the Zingiberales sequences, but nowhere else in the gene tree. **(B)** HyPhy (package FEL) site selection test. Tree shows nodes (in red) tested for positive selection. Balancing and positive selected residues are marked along the AGAMOUS protein domains. As expected, FEL detected various sites under balancing selection, while three sites were detected to be under positive selection, particularly in the I and K domains. Table shows selection test statistics for all positive selected sites observed in the analysis.

Branch selection was detected using PAML codeml branch models. Branch-selection test show significant positive selection (omega (*ω*) = 1.2059; LRT = 1,622.95153, *P* = 0.000) at the base of the Zingiberales clade, suggesting that functional divergence between lineages might have happened soon after the duplication event (Figure 5A).

**Figure 6:**
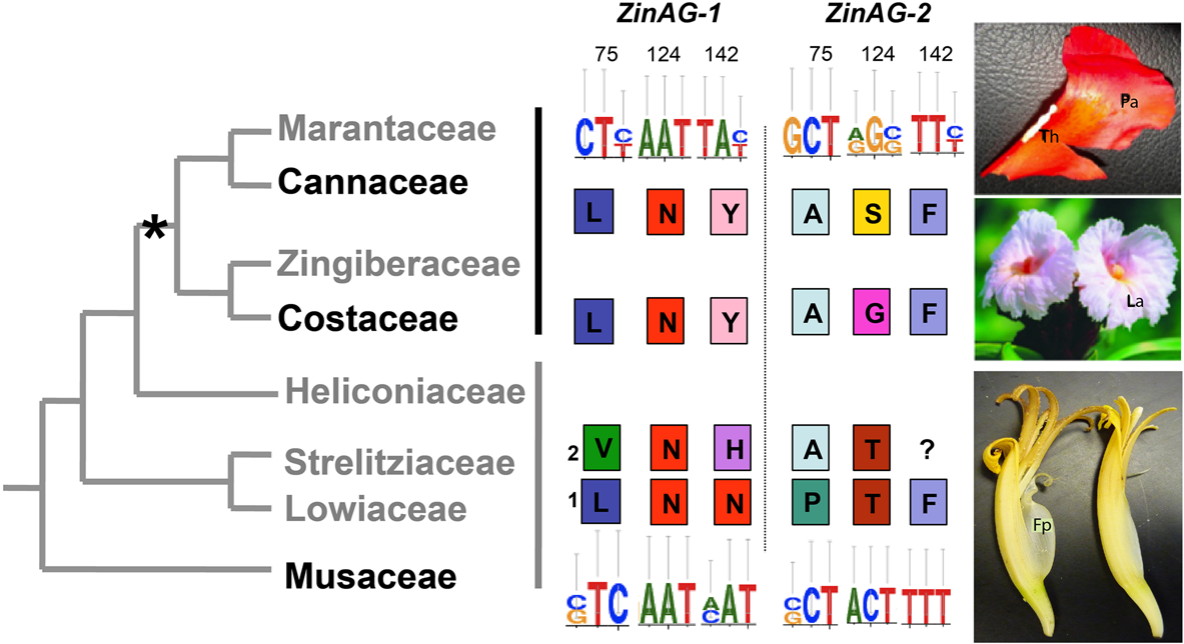
**Amino acid changes within positive selected sites for the two copies of the** ***AGAMOUS*** **(** ***AG*** **) gene across Zingiberales species.** The asterisk depicts the evolution of androecial petaloidy within the Zingiberales order. Note that it also corresponds to the base of the ginger clade (in blue). Marked in yellow are the paraphyletic lineages of the banana grade. For amino acid comparisons, *Musa acuminata* (Musaceae), *Costus spicatus* (Costaceae), and *Canna indica* (Cannaceae) *AG* sequences were used. Logos for the specific codons of the banana grade (bottom) and ginger clade (top) are shown. Single-letter amino acid traditional names were used (colored boxes). On the far right, images of *Canna indica* fertile stamen (top; Th-theca; Pa-petaloid appendage of the stamen); *Costus* sp*.* labellum (La, middle image); and *Musa basjoo* flower (bottom; Fp free petal) are shown. Also, note that *Musa acuminata* has four *AG* sequences due to a subsequent whole genome duplication event after the divergence of the Musaceae lineage [45].

Sites under selection were identified using the FEL package of HyPhy as well as site selection models of PAML *codeml*. As expected, most sites are under balancing selection, while three sites show signs of positive selection (Figure 5B). Codon position 75 in the I domain, and codon positions 124 and 142 in the K domain show signs of positive selection (Figure 5B). Comparing these sites between species of the banana grade (for example, *M. acuminata*) and the ginger group (for example, C. *spicatus* and *Canna* sp.), most of the changes, although fixed between the two groups, do not result in changes to the chemical properties of the amino acids in these positions with the single exception being the change observed at codon position 142 of *ZinAG-1* (Figure 6). In *M. acuminata*, position 142 is occupied by amino acids with charged polar side chains, such as asparagine (N) and histidine (H), while in *Canna* sp*.* and *C. spicatus* this position is occupied by tyrosine (Y), an amino acid with an uncharged side chain. Codon position 142 is part of the third alpha-helix of the K domain, also known as K3.

### Gene expression

**Figure 7:**
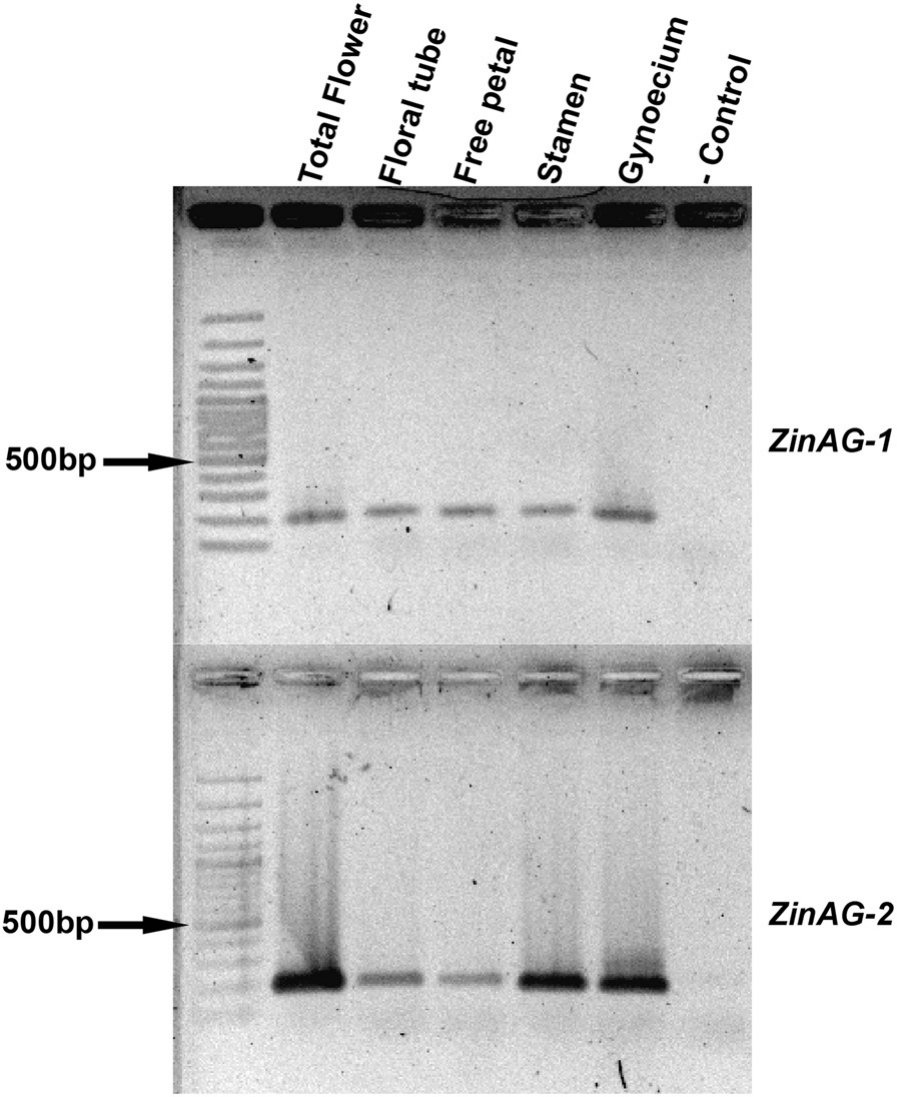
***Musa basjoo*** **RT-PCR for** ***ZinAG-1*** **and** ***ZinAG-2*** **.** RT-PCR was carried out for all *M. basjoo* floral organs, as well as for total flower cDNA as a positive control*.* RT-PCR results show expression of both copies of the gene *AG* in all floral organs studied.

*AG* expression in *M. basjoo* was initially assessed using RT-PCR (Figure 7). *ZinAG-1* and *ZinAG-2* were present in all *M. basjoo* floral organs examined.

**Figure 8:**
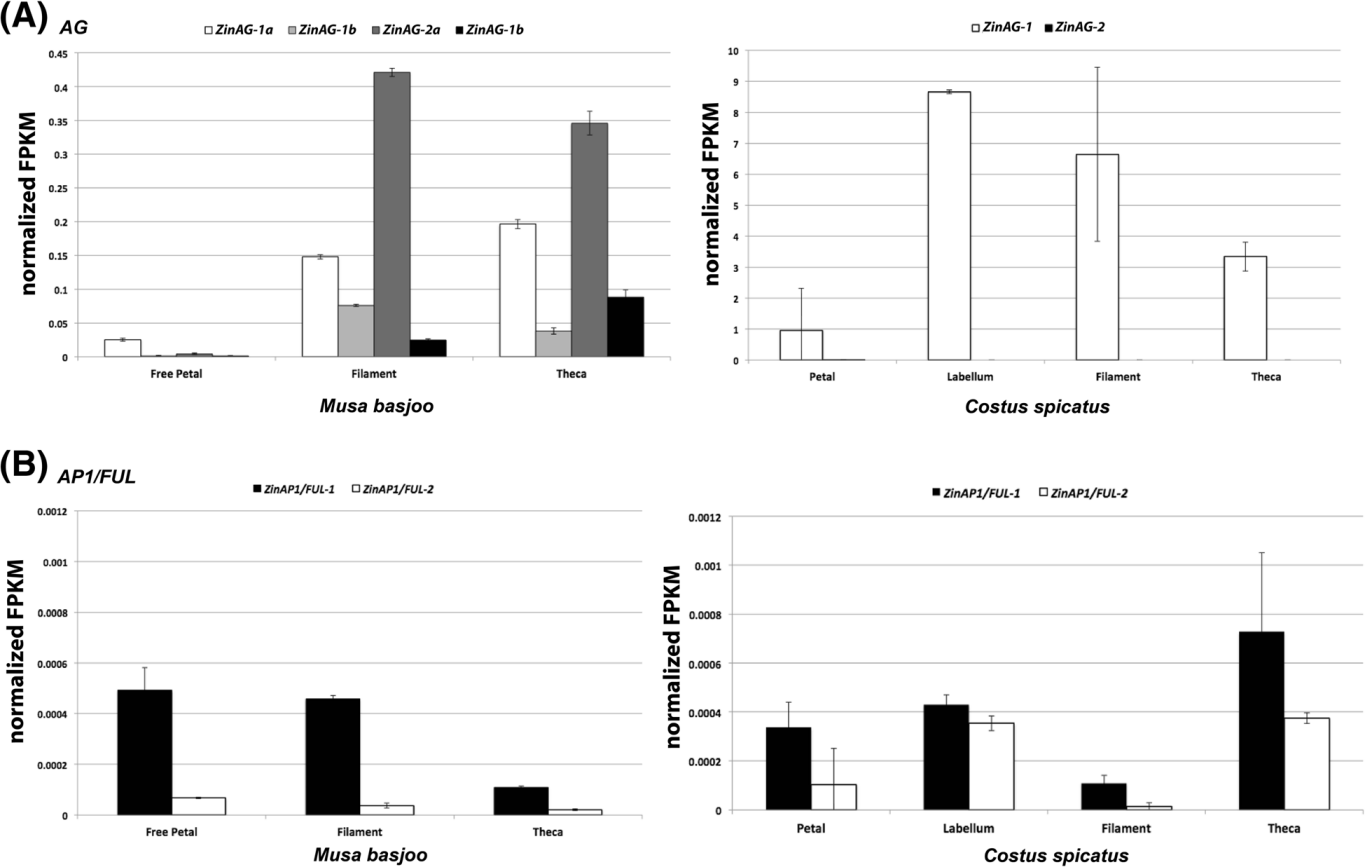
***Musa basjoo*** **and** ***Costus spicatus*** **gene expression based on transcriptomes of developing floral organs. (A)** *Musa acuminata* and *Costus spicatus AG* expression based on normalized FPKMs. *AG* has four copies in *Musa*, due to an independent duplication event. *Musa* copies are distinguished by the letters ‘a’ and ‘b,’ while ‘*ZinAG-1*’ and ‘*ZinAG-2*’ relate to the Zingiberales broad duplication event. **(B)** *Musa acuminata* and *Costus spicatus AP1/FUL-like* gene expression based on normalized FPKMs. Error bars correspond to standard deviation of two replicates.

Expression of *AG* was further investigated through organ-specific transcriptome data on *M. acuminata* and *C. spicatus* floral organs. Zin*AG-1* and *ZinAG-2* are expressed in filaments and theca of *Musa*, and in very low levels in the free petal (Figure 8A).

In the organ-specific transcriptome data, *C. spicatus AG* expression is dominated by *ZinAG-1*, and extremely low levels of *ZinAG-2* are only observed in the petal (Figure 8A), although *ZinAG-2* can be amplified by RT-PCR in these organs (data not shown). In *M. basjoo*, *AP1/FUL-like* expression largely agrees with that anticipated based on a hypothesis of mutual exclusion [46]: *APETALA1/FRUITFULL-like* (*AP1/FUL-like*) genes are mostly expressed in petals where there is very low expression of *AG*, while in stamens, *AP1/FUL-like* expression is almost abolished and *AG* is highly expressed (Figure 8B). In *C. spicatus*, *AP1/FUL-like* and *AG* gene expressions show a different pattern to that observed in *Musa*. Although *AP1/FUL-like* show very low expression values across *Costus* floral organs in comparison to *AG* expression, *AG* and *AP1/FUL-like* are simultaneously expressed in the androecial organs (labellum, stamen filament, and stamen theca), suggesting that *ZinAG-1* is not capable of fully suppressing *AP1/FUL-like* expression in these organs.

**Figure 9:**
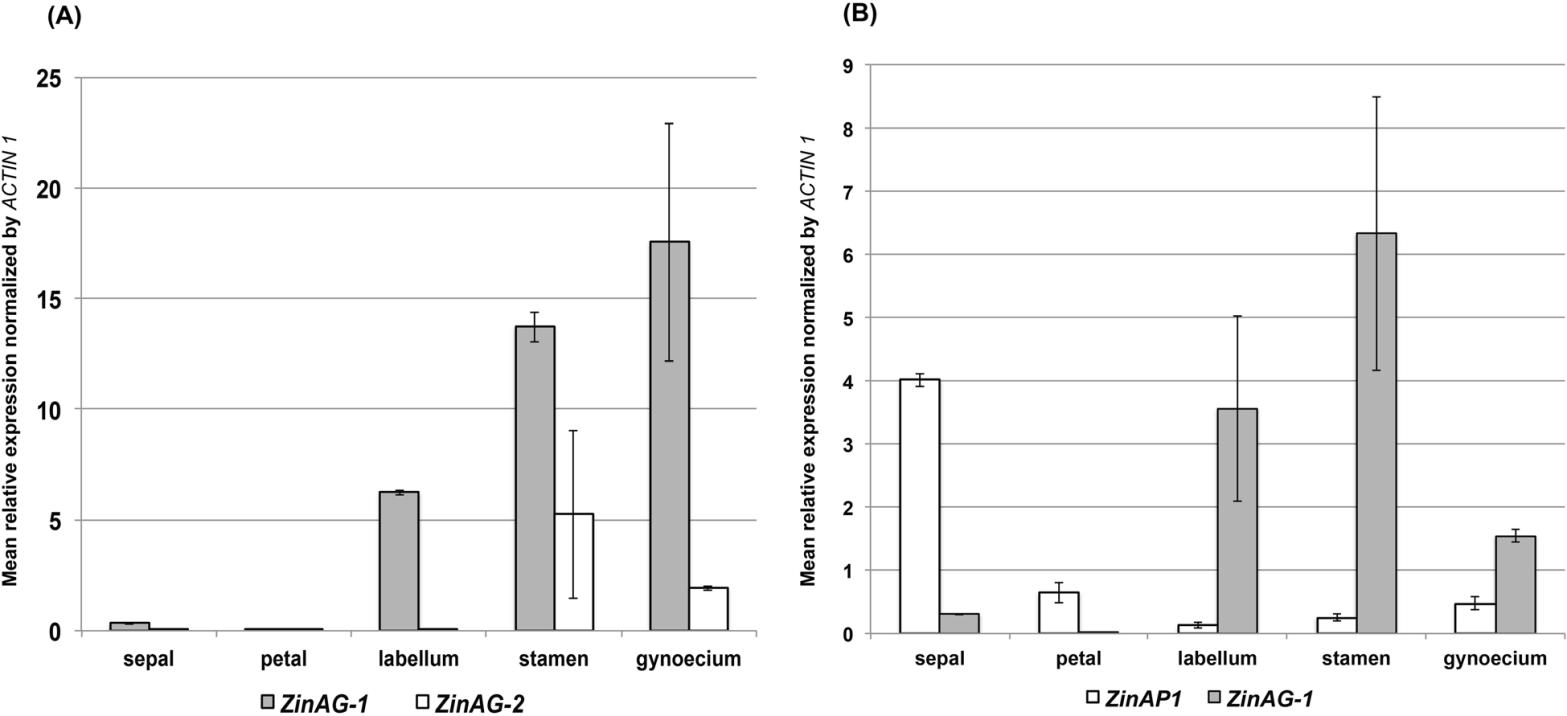
**qPCR results for** ***Costus spicatus AG*** **and** ***AP1/FUL*** **-like genes. (A)** *C. spicatus ZinAG-1* and *ZinAG-2* mean expression in all floral organs. **(B)** *C. spicatus ZinAP1* and *ZinAG-1* expression in all floral organs. Results are based on three replicates, normalized by *ACTIN1*. Error bars depict standard deviation for the three replicates.

In order to confirm transcriptome expression data, qPCR was performed in all organs of *C. spicatus* flower (sepals, petals, labellum, stamen, and gynoecium). In general, *AG* and *AP1/FUL*-like expression patterns largely agree with transcriptome data. In contrast with transcriptome data, however, *ZinAG-2* is expressed in stamen and gynoecium, with low levels of expression in labellum, petals, and sepals. The expression pattern of *ZinAG-2* agrees, in this case, with the classical expression pattern of *AG* found in model species (Figure 9A): This may indicate that *ZinAG-2* maintained the *AGAMOUS* functionality within the ginger clade. *ZinAG-1* exhibits a consistent pattern of expression between the qPCR data and the transcriptome data, showing higher levels of expression in the labellum, stamen, and gynoecium (Figure 9A). Despite its low levels when compared to *ZinAG-1*, *AP1/FUL*-like gene expression in *C. spicatus* floral organs agrees with transcriptome data (Figure 9B). In general, *AP1/FUL*-like expression can be detected in all floral organs, including stamen and gynoecium, potentially due to the inability of *AG* to fully suppress its expression in inner floral whorls (Figure 9B). It is important to notice, however, that there might be other copies of *AP1/FUL*-like genes in *C. spicatus* (as suggested by the transcriptome), and a thorough analysis of this gene family should be carried out in order to better understand the role of this gene family in flower development and morphological evolution in the Zingiberales.

## Discussion

The *AG* gene subfamily has been extensively implicated in the development of reproductive organs (carpels and stamens) and meristem determinacy in angiosperms. In both monocots and eudicots, the conservation of these functions by *AG* lineage genes is remarkable considering multiple gene duplication and subfunctionalization events [12,16,21], even though *AGL11* lineage genes might act redundantly in some lineages [10,20]. In the Zingiberales, at least one lineage-specific duplication event is observed within the *AG* lineage. Sequence divergence between the two copies (*ZinAG-1* and *ZinAG-2*), as well as their expression patterns, suggests the involvement of Zingiberales *AG* genes in the evolution of reproductive organ development and the evolution of petaloidy in the order.

Based on the branch selection patterns observed in the Zingiberales *AG* gene tree, it is likely that functional divergence between lineages happened early after the duplication event, at the base of the ginger clade. As expected for functionally important and highly interconnected genes, most of the observed site selection is due to balancing selection, suggesting functional conservation. However, three residues in the Zingiberales show signs of positive selection, and fixed differences among members of the ginger clade indicate that these modifications might be implicated in the morphological changes observed in the androecium of the Zingiberales.

In particular, the positive selected amino acid change observed at position 142 of the K domain is of particular relevance. The role of subdomains of the K domain in MADS-box protein-protein interactions has been studied, especially in the formation of dimers between B-class genes and *SEP* genes [47,48]. The K domain of the MADS proteins are involved in the formation of protein complexes for DNA binding. In particular, K1 and K2 helices are involved in dimer formation, while K3 is involved in the formation of tetramers [48-51]. Also, in *Antirrhinum*, a single amino acid change has been implicated in differences in the establishment of male and female identity between *AG* lineage genes *PLE* and *FAR*. A single glutamine insert in the K3 domain of FAR leads to a limited protein-protein interaction between AG and SEPALLATA (SEP) proteins, underlying the functional differences observed between *FAR* and *PLE* genes in determining reproductive organ identity [18].

In Zingiberales, it is possible that the amino acid change at the K3 domain observed in *ZinAG-1* between the banana grade and the ginger group might change AG protein ability to form higher level complexes while maintaining the capacity to form protein dimers. This suggests an interesting mechanism in which *ZinAG-1* from the ginger group could act as a negative regulator of tetramer formation: while binding to *AG* interacting proteins to form dimers, this complex would be less likely involved in the formation of quartets, resulting in a post-transcriptional downregulation of *AG* downstream targets.

If one assumes that *ZinAG-1* in the ginger clade (exemplified by *Costus*) inhibits quartet formation, and thus its expression leads to the downregulation of downstream targets in *C. spicatus* (as suggested by the amino acid change; Figure 6), we expect that high levels of *Costus ZinAG-1* would lead to a stronger suppression of downstream genes, and a more petal-like phenotype in the stamen whorl. The correlation between higher levels of *ZinAG-1* in *Costus* labellum and filament and a petaloid phenotype of these organs is consistent with increased levels of *ZinAG-1* in the labellum and filament and decreasing levels of *ZinAG-1* expression towards the fertile theca.

This interpretation is also supported by changes in the expression profile of *AP1/FUL-like* genes across floral organs of the Zingiberales. In *A. thaliana*, relatively high levels of *AP1/FUL* were detected in petaloid stamens and sepaloid carpels of flowers with reduced levels of *AG* due to anti-sense (RNAi) knockdown [23]. Accordingly, petaloid organs in the androecium such as those observed in *C. spicatus* are characterized by simultaneous expression of *AG* and *AP1/FUL-like*, indicating a lack of negative interaction between these two gene families. Morphologically, this expression profile corresponds to a ‘hybrid’ organ (petaloid staminode) and could potentially represent a ‘mix-attractor’ between stamen and petal in the *A. thaliana* FOS-GRN ([8], modified in Figure 1). However, this ‘hybrid’ attractor has not been observed as a stable state of the *A. thaliana* FOS-GRN, potentially due to the presumed fixed mutual negative regulation between *AG* and *AP1/FUL*. It is possible that duplication of many of the FOS-GRN genes observed in the Zingiberales could lead to stable states that are not observed in *Arabidopsis,* as different lineage-specific duplication events and subsequent differential retention/loss of duplicated copies as well as sequence divergence would provide the opportunity for novel protein interactions leading to novel stable states.

Such novel interactions are suggested by data from other monocot lineages, where an expanded *AP1/FUL-like* expression pattern has been observed in various grass lineages [52,53]. Interestingly, in *Z. mays*, constitutive expression of one of the *AP1/FUL-like* copies (*ZmFUL2a*) leads to the development of undifferentiated floral organs in the male spikelet. The authors propose an ‘interference hypothesis’ where interference of *AP1/FUL-like* proteins in the formation of proper protein-protein interactions during particular stages of development could result in the observed phenotypes [54]. Although the precise function of *A. thaliana AP1/FUL* gene might be specific to *Arabidopsis* and closely related species, studies in grasses support the idea that *AP1/FUL-like* genes do play a role in transition to flowering, meristem and perianth identity, or even in determining the identity of all floral organs ([53] and references therein).

It is important to note that the results on *AP1/FUL*-like genes presented here are preliminary. Although there is an unexpected expansion of *AP1/FUL*-like gene expression towards the inner flower whorls, a more in-depth analysis of this gene family within the Zingiberales, as well as a comprehensive survey of the expression patterns of *AG* downstream genes, is required to fully test our hypothesized scenario. Also, protein-protein interaction studies are critical to test the functions of the described *AG* protein modifications observed across the Zingiberales.

In transgenic *Arabidopsis* plants carrying *AG* anti-sense RNA, a range of floral organ phenotypes is observed including the occurrence of petaloid stamens [23]. Likewise, mutations in the regulatory site of *AG* in *Arabidopsis* can lead to the development of petaloidy in the androecium [22]. Here, we show that androecial petaloidy in the Zingiberales is likewise associated with evolution of the *AG* lineage, and may result from a single amino acid change in the K domain of *ZinAG-1* after the divergence of the banana lineages and the ginger clade.

## Conclusions

The results presented here suggest a scenario in which positive selection acting upon *AG* genes in the Zingiberales has resulted in a fixed change in the K3 domain that can potentially explain the evolution of androecial petaloidy and infertility observed in the order. Selected amino acid changes in the K3 domain might result in differential abilities to form higher level protein-protein complexes between *ZinAG-1* and its interaction partners, resulting in a post-translational downregulation of downstream genes. While further studies are needed to fully test this hypothesis, our expression data are consistent with this model. If it is the case that the changes in the *AG* genes are responsible for the observed changes in floral morphology across Zingiberales, a clear trade-off between production of fertile stamens and increased petaloidy has been fixed by positive selection in this group. Although androecial petaloidy is a remarkable feature of Zingiberales floral evolution, no changes have been observed in meristem determinacy. This might be explained, at least in part, by the potential functional redundancy between *AG* and *AGL11* lineage genes, as already reported for in rice and petunia [10,20]. Further studies of the *AG* subfamily genes in the Zingiberales will help understand the complete role of the *AG* subfamily in floral development and evolution across the Zingiberales.

## Abbreviations

*AG*: AGAMOUS
*AGL11*: *AGAMOUS-like 11*
*AP1/FUL*: *APETALA1/FRUITFULL*
*AP2*: *APETALA2*
*AP3*: *APETALA3*
BS: bootstrap support
*CsAGcp*: *Costus spicatus AGAMOUS* copy 1
*CsAGcp2*: *Costus spicatus AGAMOUS* copy 2
*CsAP1/FUL-like*: *Costus spicatus APETALA1/FRUITFULL-like* gene
*CpAG1*: *Cyclamen persicum AGAMOUS* copy 1
*CpAG2*: *Cyclamen persicum AGAMOUS* copy 2
DN: ratio of non-synonymous substitution rates
DS: ratio of synonymous substitution rates
*euAG*: *eudicot AGAMOUS*
*FAR*: *FARINELLI*
*FBP6*: Floral binding protein gene 6
FEL: fixed effect likelihood model
FOS-GRN: floral organ specification gene regulatory network
FPKM: frequency per kilobase of exon per million aligned reads
H: histidine
K domain: keratin domain
ML: maximum likelihood
*MbAGcp1*: *Musa basjoo AGAMOUS* copy 1
*MbAGcp2*: *Musa basjoo AGAMOUS* copy 2
MSA: multiple sequence alignment
N: asparagine
*OSMADS3*: *Oryza sativa MADS-box* gene 3
*OSMADS13*: *Oryza sativa MADS-box* gene 13
*OSMADS58*: *Oryza sativa MADS-box* gene 58
*PI*: *PISTALLATA*
*PLE*: *PLENA*
*PMADS3*: *Petunia MADS-box* gene 3
PP: posterior probability support
*SE*: *SEPALLATA*
SD: standard deviation
SH: Shimodaira-Hasegawa test
Y: tyrosine
*ZAG1*: *Zea mays AGAMOUS 1*
*ZinAG-1*: Zingiberales *AGAMOUS* copy 1
*ZinAG-2*: Zingiberales *AGAMOUS* copy 2
*ZMM2*: *Zea mays MADS-box gene 2*
